# Overexpression of *Atriplex canescens* Flavanone 3-Hydroxylase (*AcF3H*) Enhances Salt and Drought Tolerance in *Arabidopsis thaliana* via Flavonoid-Mediated ROS Homeostasis

**DOI:** 10.3390/plants15121783

**Published:** 2026-06-09

**Authors:** Yu-Ting Yao, Shan Feng, Bei-Bei Wang, Ai-Ke Bao

**Affiliations:** State Key Laboratory of Herbage Improvement and Grassland Agro-Ecosystems, College of Pastoral Agriculture Science and Technology, Lanzhou University, Lanzhou 730000, China

**Keywords:** *Atriplex canescens*, flavonoids, F3H, *Arabidopsis thaliana*, salt and drought stress

## Abstract

Flavonoids play critical roles in plant adaptation to abiotic stress by acting as potent antioxidants that regulate reactive oxygen species (ROS) homeostasis. In *Atriplex canescens* (Pursh) Nutt., a halophytic shrub well-adapted to saline and arid environments, transcriptomic analyses revealed that salt stress induces strong upregulation of flavanone 3-hydroxylase (F3H), a key enzyme in the flavonoid biosynthetic pathway. However, the functional role of *AcF3H* in stress adaptation remains poorly understood. Here, we cloned the *AcF3H* gene from *A. canescens* and generated transgenic *Arabidopsis thaliana* (L.) Heynh. lines constitutively overexpressing this gene. Overexpression of *AcF3H* significantly enhanced flavonoid accumulation, as confirmed by DPBA staining and total flavonoid quantification, and selectively upregulated the expression of downstream biosynthetic genes *AcDFR* and *AcANS*, which encode the dihydroflavonol 4-reductase (DFR) and anthocyanidin synthase (ANS), respectively. Under salt and drought stress, transgenic lines exhibited improved root elongation, increased shoot and root biomass, and higher relative water content compared to wild-type plants. Mechanistic investigations revealed that *AcF3H* overexpression led to reduced H_2_O_2_ accumulation and lower plasma membrane permeability under stress conditions, indicating enhanced antioxidative capacity and cellular membrane stability. These results suggest that *AcF3H* confers enhanced tolerance to abiotic stresses by promoting flavonoid-mediated ROS homeostasis. Our findings highlight *AcF3H* as a promising genetic target for engineering salt- and drought-tolerant crops.

## 1. Introduction

The distribution and productivity of plant species are predominantly determined by abiotic factors [1,2]. In response to these environmental pressures, plants are frequently subjected to various abiotic stresses, such as drought, salinity, and extreme temperatures, which severely hinder their growth and development [3,4]. Among these stresses, drought and salinity are particularly detrimental, as they significantly reduce the availability of arable land and threaten global agricultural productivity [5,6]. Therefore, elucidating the mechanisms underlying plant adaptation to these environmental challenges is essential for ensuring food security and promoting sustainable agricultural development [7,8].

*Atriplex canescens* (Pursh) Nutt., a C_4_ semi-evergreen shrub widely distributed in saline and arid regions around the globe, is recognized for its remarkable tolerance to multiple abiotic stresses, including salinity, drought, low temperatures, and heavy metal contamination, and thus is a potential horticultural plant resource for constructing a windbreak belt and greening landscapes in dry and saline regions [9,10,11,12]. Recent studies have revealed that exposure to NaCl induces a tissue-specific accumulation of flavonoids in both leaves and roots of *A. canescens*, along with a significant upregulation of the gene encoding flavanone 3-hydroxylase (F3H), a key enzyme in flavonoid biosynthesis [13,14]. These observations suggest that F3H-mediated flavonoid biosynthesis may represent a core component of the plant’s adaptive response to abiotic stress.

Flavonoids, a diverse group of polyphenolic secondary metabolites, function as potent reactive oxygen species (ROS) scavengers [15,16,17]. In addition to non-enzymatic antioxidants such as flavonoids, plants also rely on enzymatic scavengers, including superoxide dismutase (SOD), catalase (CAT), and ascorbate peroxidase (APX), to maintain ROS homeostasis [18]. These enzymes constitute the first line of defense against stress-induced oxidative damage and are distributed across different subcellular compartments (cytosol, mitochondria, chloroplasts), coordinating to protect both above-ground organs and roots from oxidative injury. Under abiotic stress conditions, particularly drought and salinity, which are leading factors in soil degradation and crop yield reduction [6], excessive ROS accumulation poses a serious threat to plant cells by damaging proteins, lipids, and nucleic acids, potentially triggering programmed cell death [15,18,19,20,21,22,23]. To mitigate oxidative damage, plants have evolved complex antioxidant defense systems, comprising both enzymatic components and non-enzymatic metabolites such as flavonoids [15,24,25]. Increasing evidence indicates that flavonoid biosynthesis is integral to the plant stress response network, mediating tolerance to a wide range of abiotic and biotic stresses [26,27,28].

Within the phenylpropanoid pathway, F3H catalyzes hydroxylation of naringenin to produce dihydroflavonols, precursors of anthocyanins and proanthocyanidins, both of which are strongly implicated in stress adaptation [29,30,31,32]. Supporting its functional relevance, ectopic expression of *F3H* from *Camellia sinensis* enhanced salt tolerance in transgenic tobacco by reinforcing antioxidant defenses [33], while expression of the moss-derived *PnF3H* gene conferred similar salt tolerance in *Arabidopsis thaliana* (L.) [34]. Despite these advances, the precise mechanisms by which *AcF3H*-mediated flavonoid biosynthesis contributes to stress resilience in *A. canescens* remain largely unexplored.

In this study, we aimed to elucidate the role of *AcF3H* in the regulation of flavonoid biosynthesis and its contribution to salt and drought stress tolerance. We hypothesized that *AcF3H* functions as a positive regulator of flavonoid biosynthesis, and that its overexpression would enhance flavonoid accumulation, thereby reducing ROS levels and improving membrane integrity under salt and drought stress. To test this hypothesis, we cloned the *AcF3H* gene from *A. canescens* and generated transgenic Arabidopsis plants overexpressing this gene. Through a combination of physiological, biochemical, and molecular methodologies, we assessed the effects of *AcF3H* overexpression on flavonoid accumulation, antioxidant capacity, and stress tolerance of transgenic plants. Our findings offer valuable insights into the molecular basis of extremophyte adaptation and propose *AcF3H* as a promising candidate gene for improving abiotic stress resistance in crop plants.

## 2. Results

### 2.1. Isolation and Characterization of AcF3H

**Figure 1:**
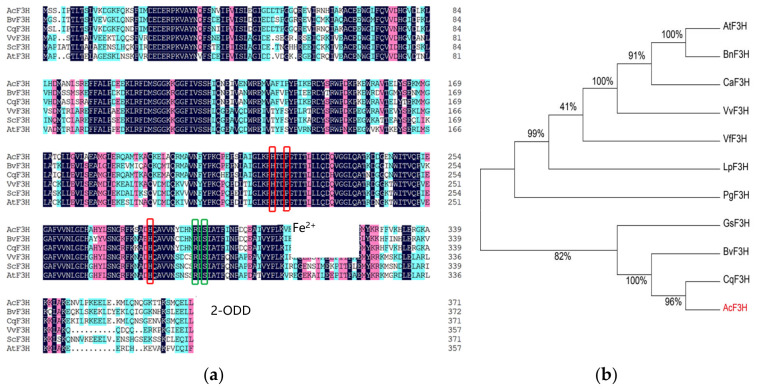
Bioinformatic analysis of *AcF3H*. (**a**) Multiple sequence alignment of AcF3H with F3H homologs from other plant species. Conserved amino acids are marked by color boxes. (**b**) Phylogenetic tree showing the relationship of AcF3H with homologous proteins from representative plant species. The tree was constructed using the neighbor-joining method in MEGA 7.0. Abbreviation: Ac, *Atriplex canescens*; Cq, *Chenopodium quinoa*; At, *Arabidopsis thaliana*; Bv, *Bauhinia variegata*; Pg, *Punica granatum*; Vv, *Vitis vinifera*; Ca, *Camptotheca acuminata*; Vf, *Vernicia fordii*; Bn, *Brassica napus*; Gs, *Glycine soja*; Lp, *Lolium perenne*; Sc, *Saccharum* spp.

The full-length coding sequence of *AcF3H* (Gene ID: Unigene9407_All), previously identified in our transcriptomic dataset [13], comprises 1119 base pairs and encodes a protein of 361 amino acids. Conserved domain analysis revealed that the AcF3H protein contains characteristic Fe^2+^-binding and 2-oxoglutarate-dependent dioxygenase (2-ODD) domains, which are essential for its enzymatic function (Figure 1a). Phylogenetic analysis further demonstrated that AcF3H clusters closely with F3H proteins from *Chenopodium quinoa*, sharing approximately 90% amino acid identity, thereby indicating strong evolutionary conservation within the *Amaranthaceae* family (Figure 1b).

### 2.2. Overexpression of AcF3H Enhanced Flavonoid Biosynthesis in Arabidopsis

**Figure 2:**
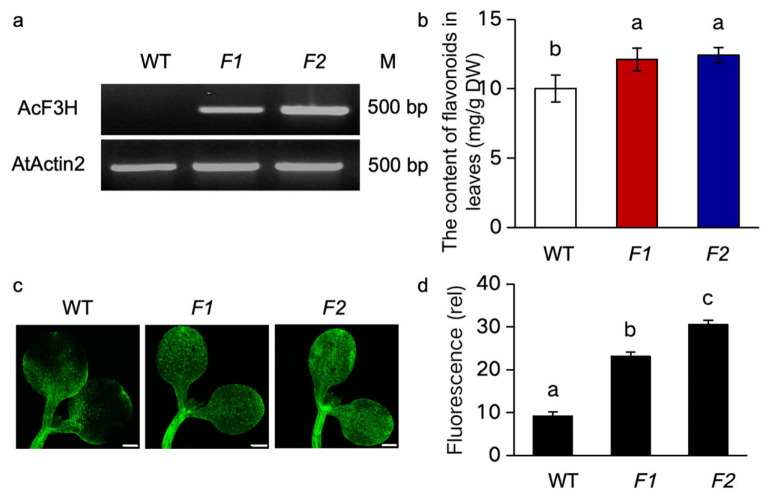
Expression analyses of *AcF3H* and flavonoid accumulation in *AcF3H*-overexpressing Arabidopsis. (**a**) RT-PCR analysis confirming *AcF3H* expression in transgenic lines *F1* and *F2*. M, DNA ladder. (**b**) Total flavonoid content in the shoots of 4-week-old WT, *F1*, and *F2* lines. Different letters above columns indicate statistically significant differences (*p* < 0.05, *n* = 6 biological replicates). (**c**) DPBA (diphenylboric acid 2-aminoethyl ester) staining of cotyledons from 6-day-old seedlings of WT, *F1*, and *F2*. Scale bars = 400 μm. (**d**) Quantification of DPBA fluorescence in *AcF3H* transgenic Arabidopsis. Different letters above columns indicate statistically significant differences (*p* < 0.05, *n* = 6 biological replicates).

To investigate the functional role of *AcF3H*, we generated transgenic Arabidopsis lines overexpressing the gene under the control of the CaMV 35S promoter using Agrobacterium-mediated transformation. Following selection and confirmation of homozygous T_3_ lines, two independent lines exhibiting differential expression, designated *F1* (moderate overexpression) and *F2* (high overexpression), were selected for further analyses (Figure 2a).

DPBA staining of 6-day-old seedlings revealed that both *F1* and *F2* lines exhibited markedly stronger fluorescence signals in leaf tissues compared to WT plants, indicating enhanced flavonoid accumulation (Figure 2). Quantitative measurements confirmed a significant increase in total flavonoid content in the shoots of *F1* and *F2* lines by 20.9% and 24.0%, respectively, relative to WT (Figure 2b).

### 2.3. Overexpression of AcF3H Upregulated Genes Involved in the Flavonoid Biosynthesis Pathway

**Figure 3:**
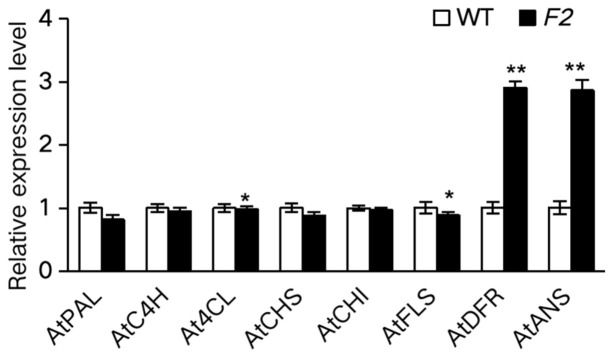
Relative expression levels of key flavonoid biosynthetic genes in the leaves of 3-week-old hydroponic WT and *F2* plants, ** represents *p* < 0.01, * represents *p* < 0.05 (Student’s *t*-test, *n* = 3 biological replicates with three technical replicates each). PAL: phenylalanine ammonia-lyase; C4H: cinnamate 4-hydroxylase; 4CL: 4-coumarate-CoA ligase; CHS: chalcone synthase; CHI: chalcone isomerase; FLS: flavonol synthase; DFR: dihydroflavonol-4-reductase and ANS: anthocyanidin synthase.

To elucidate the molecular basis underlying increased flavonoid accumulation, we analyzed the expression of eight key genes involved in the flavonoid biosynthetic pathway under normal growth conditions. Given its higher *AcF3H* expression and more pronounced phenotypic responses, the *F2* line was selected as a representative line for this analysis (Figure 3). In the *F2* line, transcript levels of the late biosynthetic genes encoding dihydroflavonol 4-reductase (DFR) and anthocyanidin synthase (ANS) were upregulated by approximately threefold compared to WT. The expression of the phenylalanine ammonia lyase (PAL) gene was moderately reduced by 20%, while the transcript levels of genes encoding other upstream enzymes, such as C4H, 4CL, and CHS, remained unchanged. These results suggest that *AcF3H* overexpression selectively enhances the expression of downstream genes in the flavonoid pathway, thereby promoting flavonoid biosynthesis.

### 2.4. Overexpression of AcF3H Improved Salt Tolerance in Transgenic Arabidopsis

**Figure 4:**
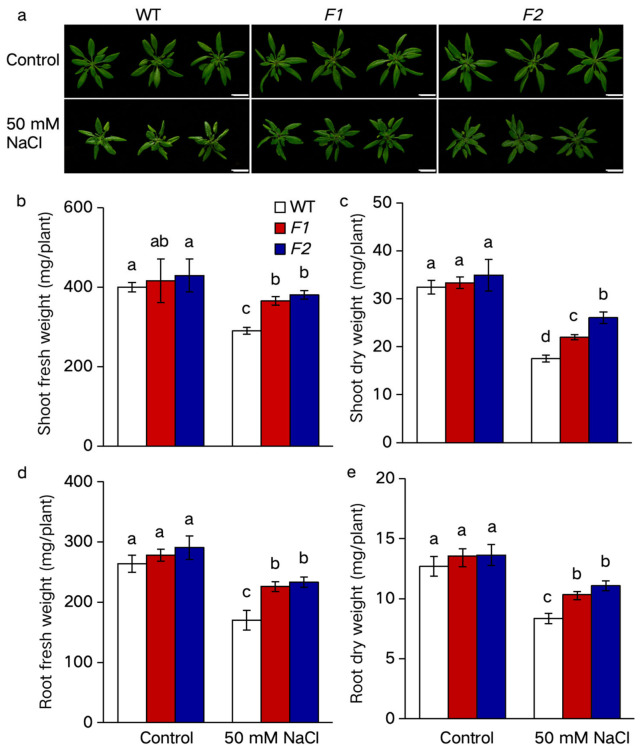
Phenotypic analysis of WT and *AcF3H*-overexpressing plants under salt stress. (**a**) Growth phenotypes of 3-week-old hydroponics WT and *F1* and *F2* plants under 50 mM NaCl treatment for 7 days. Scale bars = 2 cm. (**b**,**c**) Fresh weight and dry weight of shoots, respectively; (**d**,**e**) Fresh weight and dry weight of roots, respectively. Different letters on columns indicate statistically significant differences (*p* < 0.05, *n* = 6 biological replicates).

To determine whether *AcF3H* contributes to salt stress tolerance, we subjected WT and transgenic lines to NaCl treatment. Under standard hydroponic conditions, no phenotypic differences were observed among the genotypes. However, following 7 days of treatment with 50 mM NaCl, WT plants exhibited severe leaf curling and chlorosis, whereas *F1* and *F2* lines maintained healthier foliage and overall morphology (Figure 4a). Consistently, root elongation assays on 1/2 MS plates supplemented with 100 mM NaCl demonstrated longer primary roots in transgenic lines compared to WT (Figure S1a,b). Biomass measurements revealed that under salt stress, shoot fresh weight increased by 26.0% in *F1* and 53.3% in *F2*, while root fresh weight increased by 26.0% and 32.5%, respectively, compared to WT (Figure 4b–e). These findings confirm that *AcF3H* overexpression enhanced growth performance under saline conditions.

**Figure 5:**
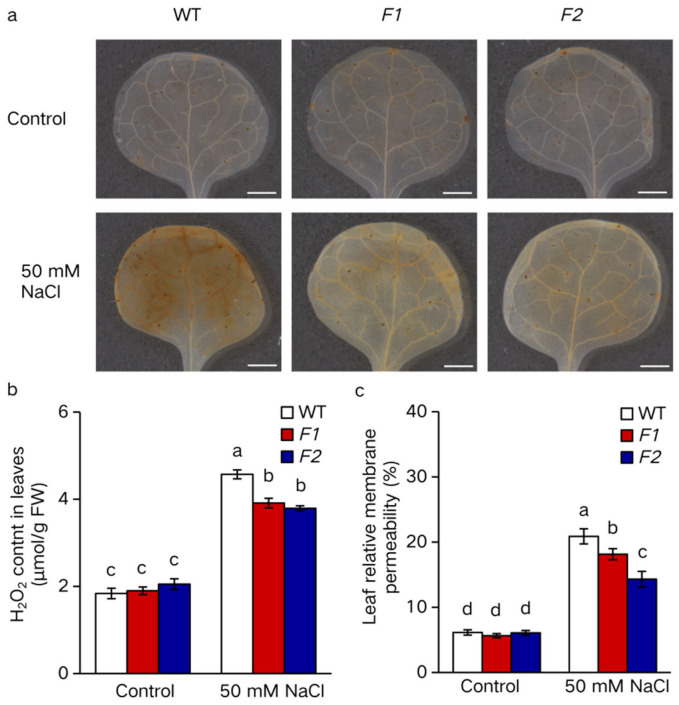
The H_2_O_2_ content and membrane permeability under NaCl treatment. (**a**) DAB staining of H_2_O_2_ accumulation in leaves of 3-week-old hydroponic WT and transgenic lines (*F1* and *F2*) under 50 mM NaCl treatment for 7 days, scale bars = 400 μm. (**b**) Quantification of H_2_O_2_ in leaves of 3-week-old hydroponic WT and transgenic lines (*F1* and *F2*) under 50 mM NaCl treatment for 3 days. (**c**) Relative plasma membrane permeability in leaves of 3-week-old plants under 50 mM NaCl treatment for 7 days. Different letters on columns represent statistically significant differences (*p* < 0.05, *n* = 6 biological replicates).

To investigate the underlying mechanism, we evaluated ROS accumulation by DAB staining and H_2_O_2_ quantification. After 7 days of 50 mM NaCl treatment, DAB staining revealed reduced H_2_O_2_ accumulation in transgenic lines compared to WT (Figure 5a). Quantitative analysis further showed that leaf H_2_O_2_ content decreased by 14.4% in *F1* and 17.1% in *F2* relative to WT (Figure 5b). In addition, relative plasma membrane permeability, a proxy for membrane damage, was significantly lower in transgenic lines (13.3% in *F1* and 31.6% in *F2*) than in WT (Figure 5c). These results suggest that *AcF3H* overexpression enhances salt tolerance by reducing oxidative damage and improving membrane stability.

### 2.5. Overexpression of AcF3H Conferred the Tolerance to Drought Stress in Transgenic Arabidopsis

**Figure 6:**
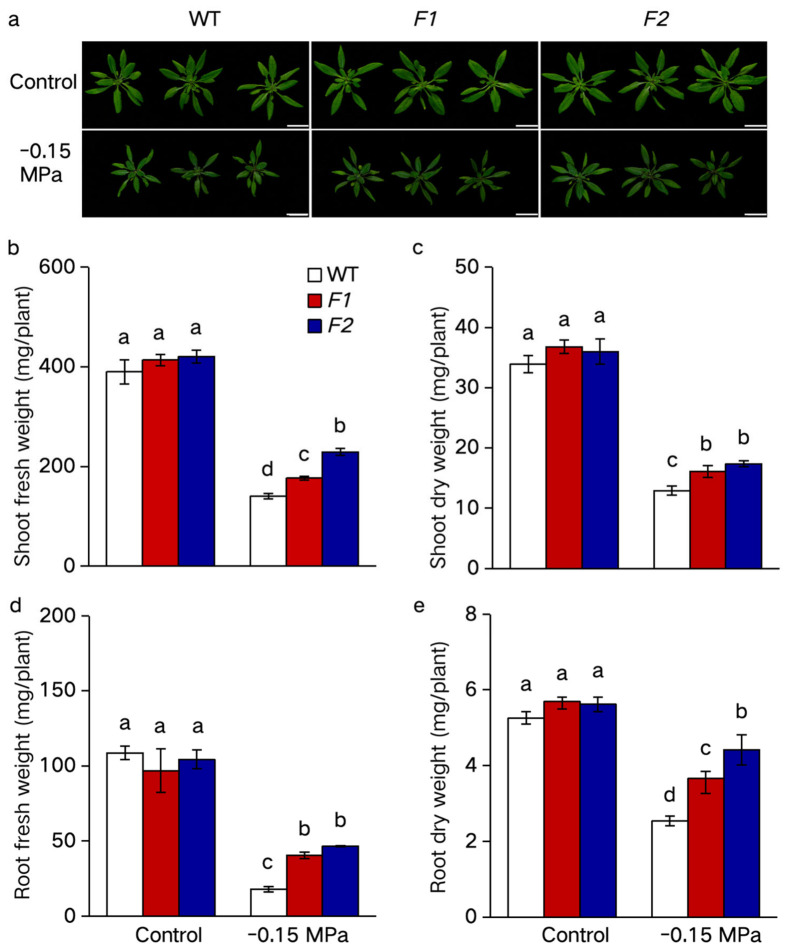
Growth performance of WT and transgenic plants under osmotic stress. (**a**) Phenotypes of 3-week-old hydroponic WT and *F1* and *F2* under 150 mM Sorbitol (−0.15 MPa) for 3 days. Scale bars = 2 cm. (**b**,**c**) Fresh weight and dry weight of shoots, respectively. (**d**,**e**) Fresh weight and dry weight of roots, respectively. Different letters on columns represent statistically significant differences (*p* < 0.05, *n* = 6 biological replicates).

To evaluate the function of *AcF3H* in enhancing drought tolerance in plants, we compared the performance of WT and transgenic lines on 1/2 MS agar plates supplemented with 200 mM sorbitol, as well as in a 1/2 strength Hoagland nutrient solution supplemented with 150 mM sorbitol (−0.15 MPa). Under osmotic stress conditions (−0.15 MPa), the transgenic plants maintained superior growth phenotypes compared to WT, with root elongation assays on 200 mM sorbitol indicating increases of 25.7% (*F1*) and 63.2% (*F2*) in root length (Figure 6a and Supplementary Figure S1c,d). In hydroponic experiments using 150 mM sorbitol, both shoot and root biomass were significantly greater in transgenic lines than in WT plants by 25% and 59%, respectively (Figure 6b–e).

**Figure 7:**
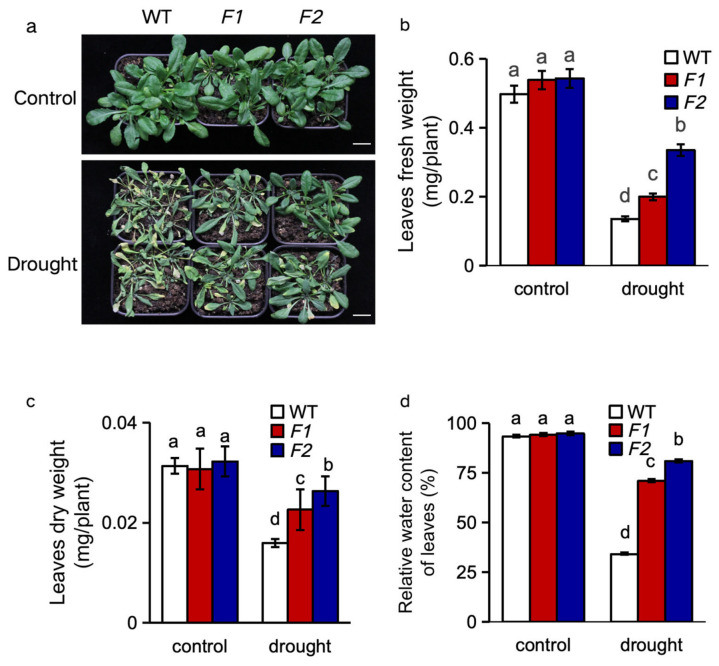
Phenotypes of WT and transgenic plants under drought treatment. (**a**) Phenotypes of 4-week-old WT and transgenic lines (*F1* and *F2*) after 14 days of drought treatment by withholding water. Scale bars = 1.5 cm. (**b**,**c**) Fresh weight and dry weight of leaves, respectively. Different letters on columns indicate statistically significant differences (*p* < 0.05, *n* = 6 biological replicates). (**d**) Relative water content (RWC) of rosette leaves after 7 days of drought stress. Different letters on columns represent statistically significant differences (*p* < 0.05, *n* = 8 biological replicates).

In soil-based drought experiments, transgenic plants displayed delayed wilting and retained turgor compared to WT under water-deficit conditions (Figure 7a). Measurements of relative water content (RWC) showed significantly higher values in transgenic leaves, indicating improved water retention capacity (Figure 7d). Consistent with this, the fresh and dry weights of the leaves of the transgenic lines were also significantly higher than those of WT plants after the drought treatment (Figure 7b,c), further confirming their enhanced growth performance under water-deficit conditions.

**Figure 8:**
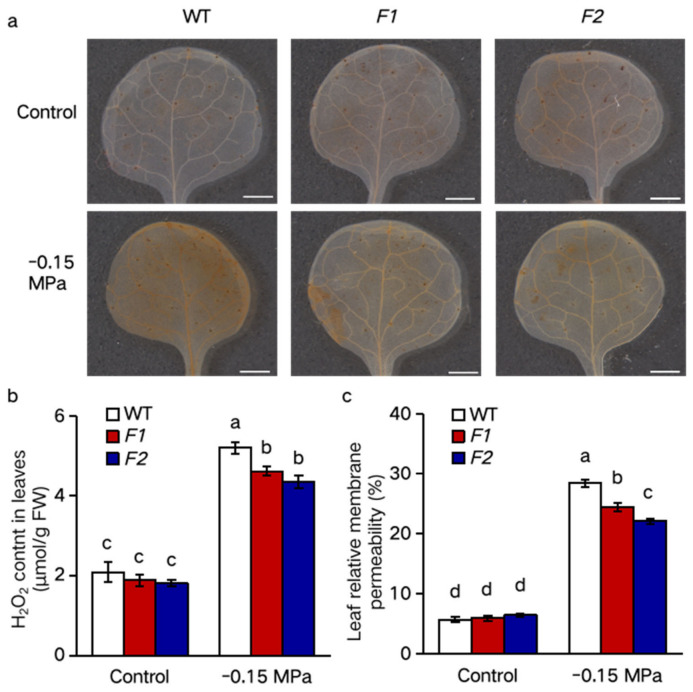
H_2_O_2_ content and relative plasma membrane permeability under drought stress. (**a**) The photographs of DAB staining of H_2_O_2_ in leaves of 3-week-old hydroponic WT and transgenic plants (*F1* and *F2*) under 150 mM Sorbitol (−0.15 MPa) treatment for 3 days. Scale bars = 400 μm. (**b**) Quantification of H_2_O_2_ in leaves of 3-week-old WT and transgenic plants (*F1* and *F2*) under 150 mM Sorbitol (−0.15 MPa) treatment for 3 days. (**c**) The leaf relative plasma membrane permeability of 3-week-old WT and *F1* and *F2* under 150 mM Sorbitol (−0.15 MPa) treatment for 3 days. Different letters on columns represent statistically significant differences (*p* < 0.05, *n* = 6 biological replicates).

To determine whether enhanced drought tolerance was associated with ROS reduction, DAB staining was performed on leaves subjected to osmotic stress. The transgenic lines displayed weaker DAB staining than WT, indicating lower H_2_O_2_ accumulation (Figure 8a). Quantitative analysis confirmed that H_2_O_2_ content was reduced by 11.4% in *F1* and 16.8% in *F2* (Figure 8b). Relative plasma membrane permeability was also significantly reduced in the transgenic lines by 14.0% (*F1*) and 22.4% (*F2*) under drought stress (Figure 8c), suggesting improved membrane integrity. These findings indicate that overexpression of *AcF3H* enhances drought tolerance in transgenic plants by mitigating oxidative stress and preserving cellular structure.

## 3. Discussion

### 3.1. AcF3H Overexpression Enhances Flavonoid Biosynthesis in Arabidopsis

In a previous study, we reported that the *AcF3H* transcript level is significantly induced in the roots and leaves of *A. canescens* upon exposure to salinity stress [14], implicating its potential role in salt adaptation. In the present study, overexpression of *AcF3H* in Arabidopsis led to a distinct accumulation of flavonoids specifically in leaf tissues, as visualized by DPBA staining (Figure 2c). This suggests that while the transcriptional regulation of *AcF3H* might be tissue-specific in its native context, the F3H enzyme itself can effectively drive flavonoid production in photosynthetic tissues when ectopically expressed. This supports the idea that flavonoids in leaves help protect photosynthesis from oxidative stress [35].

As a key enzyme in the flavonoid biosynthetic pathway, F3H converts naringenin to dihydroflavonols, thereby directing metabolic flux toward anthocyanins and proanthocyanidins—two major classes of stress-responsive flavonoids [25,36,37,38]. Our bioinformatic analysis confirmed that *AcF3H* contains the conserved catalytic domains characteristic of plant F3H homologs (Figure 1), indicating evolutionary conservation of its function. This is further supported by the observation that salt-induced F3H upregulation occurs in diverse species [39], including tomato and the Antarctic moss *Pohlia nutans* [34,40], pointing to a conserved stress-responsive regulatory module.

A striking finding from our expression analysis is the selective upregulation of downstream genes (*DFR*, *ANS*) without concomitant increases in upstream genes (*PAL*, *C4H*, *CHS*) (Figure 3). This pattern cannot be explained simply by increased substrate supply; rather, it suggests that *AcF3H* overexpression creates a metabolic pull that specifically enhances the later steps of the pathway. We propose that the accumulation of dihydroflavonols (the product of F3H) or their downstream derivatives may act as a signal to activate *DFR* and *ANS* transcription—a form of feed-forward regulation. Such metabolite-mediated transcriptional activation has been documented in other secondary metabolic pathways [41]. In contrast, the moderate reduction in *PAL* expression may reflect feedback inhibition that prevents excessive accumulation of upstream phenolic intermediates, a homeostatic mechanism commonly observed in metabolically engineered plants.

The selective activation of late-biosynthetic genes has important functional implications. By specifically boosting the conversion of dihydroflavonols to anthocyanins and proanthocyanidins, *AcF3H* not only increases total flavonoid content but also qualitatively shifts the flavonoid profile toward more potent ROS scavengers [42,43]. This shift likely underlies the enhanced stress tolerance observed in our transgenic lines, as anthocyanins are several-fold more efficient at neutralizing ROS than many other flavonoid classes [41].

### 3.2. AcF3H Overexpression Enhances the Salt and Drought Tolerance of Transgenic Arabidopsis via Flavonoid-Mediated ROS Scavenging

Flavonoids are multifunctional metabolites with well-documented roles in modulating plant responses to abiotic stress, primarily through their antioxidant activity [35,44,45,46]. Under salt and drought stress, plants experience oxidative bursts characterized by excessive accumulation of ROS, which can damage cellular components and compromise membrane integrity [15,19,47,48]. In this study, the overexpression of *AcF3H* conferred enhanced salt and drought tolerance in Arabidopsis, as demonstrated by improved growth performance, biomass accumulation, and relative water content under stress conditions (Figure 4, Figure 6 and Figure 7). These phenotypic improvements were accompanied by a significant reduction in H_2_O_2_ levels and plasma membrane permeability in transgenic lines compared to WT (Figure 5 and Figure 8), indicating improved ROS detoxification and membrane stability.

The enhanced antioxidant capacity observed in *AcF3H*-overexpressing lines is likely attributable to the elevated flavonoid content, which not only scavenges ROS directly but may also modulate antioxidant signaling pathways such as the ascorbate-glutathione cycle [44,49]. Our findings corroborate prior studies in *P. nutans* and *C. sinensis*, where *F3H* overexpression reduced oxidative damage under salt and drought stress [33,34]. Furthermore, the reduced membrane permeability observed in transgenic plants (Figure 5c and Figure 8c) suggests that flavonoid-mediated ROS scavenging plays a protective role in maintaining membrane integrity, possibly by mitigating lipid peroxidation. This is consistent with the concept of a conserved F3H-flavonoid-ROS-membrane protection axis in stress-adaptive responses [50,51]. While our study focused on flavonoid-mediated ROS scavenging, plants possess a coordinated antioxidant network that includes both enzymatic components (SOD, CAT, APX, GR) and non-enzymatic metabolites (flavonoids, ascorbate, glutathione) to counteract oxidative stress [52]. Among these, SOD acts as the first line of defense, catalyzing the dismutation of superoxide radicals to H_2_O_2_ and O_2_, with distinct isoforms localized in the cytosol, mitochondria, and chloroplasts [53]. Since the stress conditions applied in this study affect both shoots and roots, future work should investigate the tissue-specific expression patterns of these enzymatic antioxidants and their potential interactions with flavonoid accumulation. The protective strategies in above-ground organs may differ quantitatively or qualitatively from those in roots. Elucidating how *AcF3H* overexpression modulates these organ-specific antioxidant responses will provide a more comprehensive understanding of the stress-adaptive network.

## 4. Materials and Methods

### 4.1. Plant Materials and Growth Conditions

Seeds of *A. canescens* were obtained from the USDA-ARS GRIN taxonomy database (GRIN ID: 6000), and seeds of Arabidopsis. ecotype Col-0 from the Arabidopsis Biological Resource Center (ABRC, Stock No. CS78423). The procedures for *Agrobacterium*-mediated genetic transformation via the floral dip method and seedling cultivation were performed as previously described by Feng et al. (2024) [14]. All plants were grown in a controlled growth chamber under the following standard conditions: photoperiod of 16 h light/8 h dark, light intensity of 150 µmol·m^−2^·s^−1^, temperature of 22 ± 3 °C, and relative humidity of 63 ± 2%. These conditions were used for both *A. canescens* and Arabidopsis unless otherwise specified in specific experiments.

### 4.2. Cloning of AcF3H Gene and Bioinformatics Analysis

The open reading frame (ORF) of the *AcF3H* gene was predicted using ORF Finder 2.0 (http://www.bioinformatics.org/sms2/orf_find.html (accessed on 14 May 2026)). Two pairs of nested primers (Table S1) were designed based on the gene sequence, and cDNA synthesized from *A. canescens* RNA served as the template for PCR amplification. The full-length gene was amplified using the Phusion^®^ High-Fidelity DNA Polymerase kit (Thermo Fisher Scientific, Waltham, MA, USA), following the manufacturer’s instructions and the amplification parameters established in Feng et al. (2024) [14].

The deduced amino acid sequence of AcF3H was analyzed using DNAMAN 6.0 software for multiple sequence alignment with homologous proteins. A phylogenetic tree was constructed using MEGA 7.0 software (MEGA, Center for Evolutionary Medicine and Informatics, Tempe, AZ, USA) to assess evolutionary relationships.

### 4.3. Generation of Transgenic Overexpressing AcF3H

The complete coding sequence (CDS) of *AcF3H* was amplified using primers containing *NcoI* restriction sites (Table S1) and ligated into the binary vector pCAMBIA1302-*Basta*-*35S* using the In-Fusion^®^ HD Cloning Kits (TaKaRa, Osaka, Japan). In this construct, the *AcF3H* CDS was placed under the control of the CaMV 35S promoter and followed by the NOS terminator. The orientation of the insert was verified by restriction enzyme digestion and DNA sequencing. The recombinant plasmid pCAMBIA1302-*Basta*-*35S*-*AcF3H* was introduced into *A. tumefaciens* strain GV3101 and transformed into Arabidopsis Col-0 via the floral dip method [54]. T_3_ generation homozygous lines were selected using 0.1‰ (*v*/*v*) Basta solution. Reverse transcription PCR (RT-PCR) was performed to identify lines with high *AcF3H* expression levels (Table S1). Two representative lines with differential expression, designated *F1* (moderate overexpression) and *F2* (high overexpression), were selected for downstream analyses.

### 4.4. Flavonoid Localization and Quantification

Flavonoid distribution was visualized in 6-day-old seedlings via histochemical staining using diphenylboric acid 2-aminoethyl ester (DPBA). The staining procedure was as follows: 6-day-old seedlings were immersed in 0.25% (*w*/*v*) DPBA solution (prepared in 0.005% Triton X-100) for 45 min at room temperature, then washed three times with distilled water. Fluorescence was visualized using a fluorescence microscope (Zeiss, Jena, Germany) with excitation at 488 nm and emission at 520 nm. ImageJ (v1.8.0, National Institutes of Health (NIH), Bethesda, MD, USA) software was used to quantify the mean fluorescence intensity from the DPBA-stained images. Flavonoid content was quantified by aluminum-nitrite colorimetry: 0.018 g dry weight was extracted with 1.8 mL 60% ethanol at 60 °C for 2 h, centrifuged, and the supernatant absorbance measured at 510 nm; content was calculated as 0.398 × (ΔA510 − 0.0007)/DW [14].

### 4.5. Expression Profiling of Flavonoid Pathway Genes

To investigate the effect of *AcF3H* overexpression on the flavonoid biosynthetic pathway in transgenic Arabidopsis, we assessed the relative expression levels of eight genes encoding key enzymes in flavonoid pathway by quantitative real-time PCR (qRT-PCR): phenylalanine ammonia lyase (PAL), cinnamate 4-hydroxylase (C4H) 4-coumarate-CoA ligase (4CL), chalcone synthase (CHS), flavanone 3-hydroxylase (F3H), flavonol synthase (FLS), dihydroflavonol-4-reductase (DFR) and anthocyanidin synthase (ANS). *AtActin2* was used as the internal reference gene. Total RNA was extracted using the TaKaRa Mini BEST Plant RNA Extraction Kit (TakaRa, Kusatsu, Japan), and first-strand cDNA was synthesized using the PrimeScript™ II 1st Strand cDNA Synthesis Kit (TaKaRa Biotechnology, Dalian, China). The qRT-PCR was conducted with SYBR^®^ Premix Ex Taq™ II (TaKaRa Biotechnology, Dalian, China) on a StepOnePlus real-time system (Applied Biosystems, Foster City, CA, USA). Relative gene expression levels were calculated using the 2^−ΔΔCt^ method. All reactions were performed in triplicate. Primer sequences are listed in Supplementary Table S1.

### 4.6. Phenotypic Evaluation Under Salt and Drought Stress

To assess stress tolerance, seeds of WT and transgenic lines were germinated on half-strength Murashige and Skoog (1/2 MS) medium supplemented with 1.5% (*w*/*v*) agar for 3 days. Based on preliminary experiments, 100 mM NaCl and 200 mM sorbitol were selected because they caused moderate but consistent growth inhibition in wild-type plants, allowing clear detection of both enhanced tolerance and potential suppression. Uniform seedlings were transferred to either control 1/2 MS plates or plates supplemented with 100 mM NaCl (salt stress) or 200 mM sorbitol (osmotic stress). After 10 days of growth, phenotypes were documented, and root lengths were measured using ImageJ (v1.8.0, NIH, Bethesda, MD, USA). Each experiment was independently repeated six times.

For hydroponic experiments, 3-week-old seedlings were treated with either 1/2 Hoagland solution [14] (control) or 1/2 Hoagland supplemented with 50 mM NaCl for 7 days. For drought stress, 24-day-old seedlings were exposed to 1/2 Hoagland solution containing 150 mM sorbitol (−0.15 MPa) for 3 days. These concentrations were chosen as they induced clear stress phenotypes without causing complete lethality, based on preliminary trials. Growth phenotypes were recorded, and tissues were collected for physiological analyses. To further verify the drought tolerance of the transgenic line, the uniform 2-week-old seedlings were divided into two groups: one received regular watering (control), while the other was subjected to drought stress by withholding water for 14 days. Leaf samples were harvested for physiological measurements. The phenotypic observations were documented through photography and were harvested for growth and physiological measurements.

Fresh weight (FW) of roots and shoots was recorded immediately after harvest. Samples were then dried at 80 °C for 48 h to obtain dry weight (DW). In soil-grown plants, leaf turgid weight (TW) was determined after soaking leaves in water for 8 h. Relative water content (RWC) was calculated as: RWC = (FW − DW)/(TW − DW) × 100%.

### 4.7. Measurement of Hydrogen Peroxide (H_2_O_2_) Content

To assess the oxidative damage of salt and drought stress on plants, H_2_O_2_ accumulation in leaf tissues was examined following the method of Daudi and O’Brien (2012) using diaminobenzidine (DAB) staining [55]. Leaf samples were collected from 3-week-old hydroponic WT and transgenic plants subjected to 50 mM NaCl for 7 d or 150 mM sorbitol for 3 d. For DAB staining, leaves were incubated in DAB solution (1 mg·mL^−1^, pH 3.8) for 8 h in the dark at room temperature, then destained in 95% ethanol until chlorophyll was completely removed. Stained leaves were photographed, and the intensity of brown coloration was used as an indicator of H_2_O_2_ accumulation. Quantitative determination of H_2_O_2_ levels was performed using a commercial detection kit (Suzhou Comin Biotechnology Co., Ltd., Suzhou, China) following the manufacturer’s instructions, as described in Feng et al. (2024) [14]. Each treatment was replicated six times.

### 4.8. Measurement of Plasma Membrane Permeability

Relative plasma membrane permeability was assessed as an indicator of membrane integrity under stress. Fresh leaves from hydroponically grown plants were collected following salt or drought treatment. Electrolyte leakage was measured using a conductivity meter (REX, DDSJ-319L, Shanghai, China) based on standard protocols.

### 4.9. Statistical Analysis

Statistical analyses were performed using SPSS 23.0 (IBM, Armonk, NY, USA). One-way analysis of variance (ANOVA) was used to assess significant differences among treatments. A *p*-value of less than 0.05 was considered statistically significant.

## 5. Conclusions

Our study demonstrates that overexpression of *A. canescens AcF3H* in Arabidopsis stimulates the flavonoid biosynthesis pathway, resulting in increased accumulation of stress-responsive flavonoids. This biochemical shift enhances the plant’s antioxidant capacity, thereby reducing ROS accumulation and preserving cellular membrane integrity under salt and drought stress. These findings not only elucidate the molecular function of AcF3H in abiotic stress tolerance but also underscore its potential as a genetic resource for engineering crops with improved resilience to salinity and drought.

Several limitations of this study should be acknowledged. First, the lack of a stable genetic transformation system for *A. canescens* currently prevents functional validation of *AcF3H* in its native species. Second, our analyses were confined to vegetative stages; therefore, the effects of *AcF3H* overexpression on generative development remain unknown. Third, although we demonstrated flavonoid-mediated ROS scavenging, the potential interplay between *AcF3H* and other antioxidant systems (e.g., superoxide dismutase, catalase, ascorbate peroxidase) was not examined, nor did we investigate tissue-specific responses in shoots versus roots.

Future efforts should prioritize the establishment of a transformation system for *A. canescens* to validate *AcF3H* function in its native context. In addition, comparative analyses of flavonoid accumulation and enzymatic antioxidant activities across different organs (shoots and roots) under diverse stress conditions would provide a more comprehensive understanding of the antioxidant network activated by *AcF3H* overexpression. Finally, assessing the impact of *AcF3H* on reproductive success and yield under field conditions will be essential for agricultural applications.

## Data Availability

All data presented in this study are available in the article and Supplementary Materials. Further inquiries can be directed to the corresponding author.

## Supplementary Materials

The following supporting information can be downloaded at: https://www.mdpi.com/article/10.3390/plants15121783/s1. Figure S1: (a) The growth of seedlings WT and transgenic lines (*F1* and *F2*) on 1/2 MS solid medium with 100 mM NaCl for 10 days. Scale bars = 1 cm; (b) The roots length of (a), *** represent *p* < 0.001, *n* = 9; (c) The growth of seedlings WT and transgenic lines (*F1* and *F2*) on 1/2 MS solid medium with 200 mM sorbitol for 10 days, Scale bars = 1 cm; (d) The roots length of (c), *** represent *p* < 0.001, *n* = 9; Supplementary Table S1: Primer sequences used in qRT-PCR.
