# Supplementary material for: Overexpression of Atriplex canescens Flavanone 3-Hydroxylase (AcF3H) Enhances Salt and Drought Tolerance in Arabidopsis thaliana via Flavonoid-Mediated ROS Homeostasis
Source: Plants (Basel). 2026 Jun 9;15(12):1783. doi: 10.3390/plants15121783 (PMC13307176; doi:10.3390/plants15121783)
Supplement: Supplementary file 1 [file plants-15-01783-s001.zip › plants-4350748-supplementary.pdf]

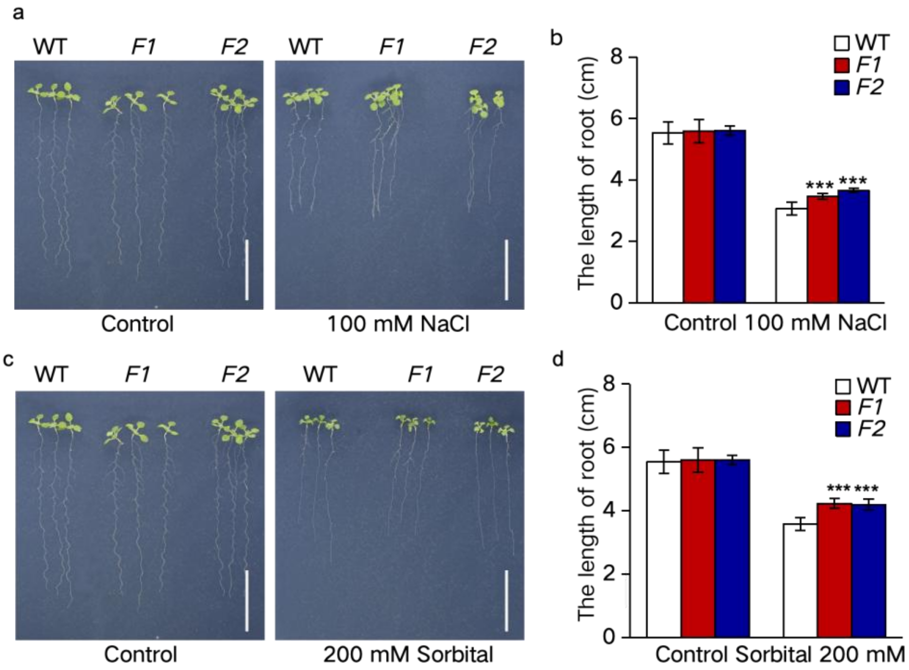

Figure S1. (a) The growth of seedlings WT and transgenic lines (*F1* and *F2*) on 1/2 MS solid medium with 100 mM NaCl for 10 days. Scale bars=1 cm; (b) The roots length of (a), *** represent $p < 0.001$,n=9; (c) The growth of seedlings WT and transgenic lines (*F1* and *F2*) on 1/2 MS solid medium with 200 mM sorbitol for 10 days, Scale bars=1 cm; (d) The roots length of (c), *** represent $p < 0.001$, n=9.

Supplementary Table S1. Primer sequences used in qRT-PCR.

Primers	Sequences (5′-3′)
Out-F	TCTATTCCTCTCACCCCTGC
Out-R	CTCACTAAATATAGCAAAG
Inner-F	GTAGTGTGTAAGATCGATC
Inner-F	GATAAAGTAGACTCGAA
AcF3H-RTF	TCTATTCCTCTCACCCCTGC
AcF3H-RTR	GAAATAGCCCCCACTCCTCG
AcActin-F	AAGAACTACGAGCTACCTGACGG
AcActin-R	GATACCAGAAGATTCCATTCCAAC
AcF3H-F	GGACTCTTGA<u>CCATGG</u>CCATGAGTTCAATTCCTAC GCTAACAT
AcF3H-R	GTCAGATCTA<u>CCATGG</u>TTAAGCAAGTAACTCTTGC

	ATGGAT
AtActin2-RT-F	TCATGCCATCCTCCGTCTTG
AtActin2-RT-R	TGAACGATTCCTGGACCTGC
AcF3H-RT-F	ATGAGTGGGGGCAAGAAAGG
AcF3H-RT-R	CCCAATAGCAAGACTGGGCT
AtActin-qRT-F	TCAGATGCCCAGAAGTGTTGTTCC
AtActin-qRT-R	CCGTACAGATCCTTCCTGATATCC
AtC4H-qRT-F	GGCTAGCAAACAACCCCAAC
AtC4H-qRT-R	GCTTCCACGTGCGATTCTTC
At4CL1-qRT-F	TCGTGTCGAAACAGGTTGTG
At4CL1-qRT-R	AGTTTTGCCCTCAGATCTTTCCT
AtPAL1-qRT-F	GCCTGGAGAAGAGTTCGACA
AtPAL1-qRT-R	CTCCGTTCCACTCGTTGAGA
AtCHS-qRT-F	CAAAGAAGCGGCAGTGAAGG
AtCHS-qRT-R	CCGGAGGTAGTGCAGAAGAC
AtF3H-qRT-F	TCTGCCGTCAGATCGTTGAG
AtF3H-qRT-R	AGCAAAGAAGTCACGAGCGA
AtFLS1-qRT-F	GCAATCCCGTTGGAGTTCATC
AtFLS1-qRT-R	CTTCGTCGGGATCGCTTAGA
AtDFR-qRT-F	CAAACGCCAAGACGCTACTC
AtDFR-qRT-R	GGAAAACACCGTCACATCCG
AtANS-qRT-F	TGGTTGCGGTTGAAAGAGTTG
AtANS-qRT-R	TCGAGCTCTTCTTTTGGACGA
AtCHI-qRT-F	GGTAACGCCGTTCCTTCTCT
AtCHI-qRT-R	ACGCACCGGTGACTATTTCA
